# Global comparison of urban bike-sharing accessibility across 40 cities

**DOI:** 10.1038/s41598-024-70706-x

**Published:** 2024-09-03

**Authors:** Sachit Mahajan, Javier Argota Sánchez-Vaquerizo

**Affiliations:** Computational Social Science, ETH Zurich, 8092 Zurich, Switzerland

**Keywords:** Civil engineering, Environmental social sciences

## Abstract

The global expansion of bike-sharing networks offers a cost-effective and environmentally friendly transportation alternative that complements public transit and promotes active, healthy lifestyles. Despite significant research, most studies focus on individual locations, specific environmental or health impacts, or infrastructure elements like bike lanes. Instead, this paper elaborates a comprehensive global comparison of bike-sharing systems by introducing a novel database that aggregates data from 40 cities worldwide. Our study integrates this data with population data and urban metrics to classify these networks topologically and assess their effective coverage concerning the population served and their relation with existing public transit systems. We introduce the “Bike-Share Service Accessibility Index” (BSAI), a new metric to evaluate and compare the performance of bike-sharing networks. Our findings provide valuable insights for urban planners and policymakers, offering data-driven strategies to enhance sustainable urban mobility through better-integrated and more spatially equitable bike-sharing systems.

## Introduction

In the evolving landscape of urban mobility, the creation of sustainable and accessible transportation systems has become a paramount concern of cities worldwide^1,2^. This drive is fueled by both, by the challenge of quickly progressing urbanization and the goal to mitigate climate change. As the urban population grows, cities face the daunting task of managing increased vehicular traffic, air pollution, and CO2 emissions, all while striving to maintain the quality of urban life^3–5^. Public bike-sharing systems have emerged as a promising solution, offering an eco-friendly alternative to traditional motorized transport and starting a new era in urban mobility^6^. While bike-sharing systems have gained popularity globally, their implementation and impact vary significantly across different urban contexts^7,8^. Several studies have explored these variations, comparing bike-sharing systems across cities and countries. For instance, in one of the works^9^, researchers analyzed 75 case studies of bike-share systems, finding major differences in system performance and highlighting the importance of system design and policy support. Similarly, another work explored the dockless bike-sharing system in Singapore and emphasized the role of land-use practices, fleet size and local contexts in shaping system characteristics and usage patterns^10^. Mateo-Babiano et al.^11^ examined bike-sharing systems in Australian cities, investigating how environmental features impact the usage of public bike-sharing system. In another work, Shi et al.^12^ exmphasized the role of stakeholder collaboration as well as public-private partnerships to achive bike-sharing sustainability in China. However, despite these valuable contributions, there remains a gap in comprehensive, standardized comparisons of station-based bike-sharing systems across a large number of global cities.

The concept of bike-sharing is not merely about providing bicycles for rent; it is an innovative approach to urban transportation that complements existing transit networks, promotes environmental sustainability, and can support healthier, more active lifestyles among urban residents^13,14^. Studies have shown that bike-sharing systems can significantly reduce reliance on private vehicles, thereby decreasing pollutants and contributing to cleaner, greener urban environments^15^. Moreover, the flexibility and convenience of bike-sharing can enhance urban accessibility, enabling residents and visitors to navigate cities more efficiently and potentially with greater autonomy^16^. In this study, we focus specifically on bike-share infrastructure-the network of stations and bicycles in public bike-sharing services. This is distinct from general cycling infrastructure like bike lanes or paths. Our analysis centers on the distribution and effectiveness of these bike-share systems as urban mobility solutions across different cities. We focus exclusively on station-based bike-sharing systems, which include both traditional docked systems and geo-fenced systems. Traditional docked systems require bikes to be returned to specific docking stations with fixed capacities. Geo-fenced systems allow bikes to be parked within designated areas without physical docks^17^.

Despite the growing popularity and benefits of bike-sharing systems, their implementation and integration into the urban fabric present a series of challenges. Central among these is the need to design and deploy bike-share infrastructures that are not only extensive but also spatially equitable and accessible to all segments of the urban population^18,19^. The issue of equity in bike-sharing systems has also been a focus of recent research. Hosford and Winters^20^ analyzed the distribution of bike-sharing stations in relation to socioeconomic factors in Canadian cities, finding disparities in access. Similarly, several works^21–23^ have examined equity considerations in bike-share system design across different cities, highlighting the need for fair and just planning to ensure equitable access.

Research has highlighted disparities in the accessibility of bike-sharing services, finding that certain areas and demographics are often under-served by existing networks^24^. Bike-sharing systems can contribute to a fair transport infrastructure^25^, although there is often a gap between theory and practice^26^ and these concerns need to considered in their design^27^. Additionally, the sustainability of bike-sharing systems hinges on their ability to integrate seamlessly with other modes of public transport, thereby creating a cohesive and comprehensive urban mobility solution^28^.

Expanding upon the existing discourse within the literature regarding bike-sharing systems reveals both the depth and breadth of research dedicated to understanding their impact and potential. Studies such as those by Fishman et al.^15^ have delved into the environmental benefits of bike-sharing, highlighting its role in reducing urban pollutants and offering a sustainable alternative to motorized transport. Similarly, research by Fuller et al.^29^ emphasizes the health benefits associated with increased physical activity through bike-sharing. In addition to environmental and health benefits, studies have explored the economic implications of bike-sharing systems^30^. For instance, DeMaio^31^ examines the cost-effectiveness of bike-sharing programs and their potential to reduce transportation costs for users. These studies underscore the multifaceted advantages of bike-sharing, from environmental sustainability to public health improvements.

However, a critical examination of the literature uncovers significant gaps, particularly regarding the comparative analysis of bike-sharing systems across different cities around the world. As Midgley^1^ articulates that the scarcity of studies exploring bike-sharing across varied geographical and cultural landscapes hinders our ability to identify best practices or adapt strategies to suit specific urban environments. This limitation is further compounded by the tendency of existing research to compartmentalize the examination of bike-sharing, often isolating specific elements such as user demographics^15^, route choices^32^, infrastructure cost^33^ or economic impacts^16^, without a comprehensive view of the systems’ integration within the broader urban mobility network. Moreover, the literature often overlooks the critical issue of accessibility and equity in bike-sharing systems. Research by Goodman and Cheshire^34^ begins to address these concerns, noting disparities in access to bike-sharing services among different socio-economic groups and calling for a more inclusive approach to bike-sharing infrastructure development. Yet, comprehensive studies that link these accessibility concerns with broader analyses of bike-sharing efficacy and efficiency are notably lacking.

Addressing these gaps, our study introduces a novel approach by focusing on the infrastructure of bike-sharing systems, specifically the strategic placement and accessibility of bike stations, rather than the extensively studied subjects of bike lanes and other cycling-related infrastructures^35–37^. This focus allows us to explore a critical but often overlooked aspect of bike-sharing systems that plays a vital role for their effectiveness, accessibility, and user adoption.

**Fig. 1:**
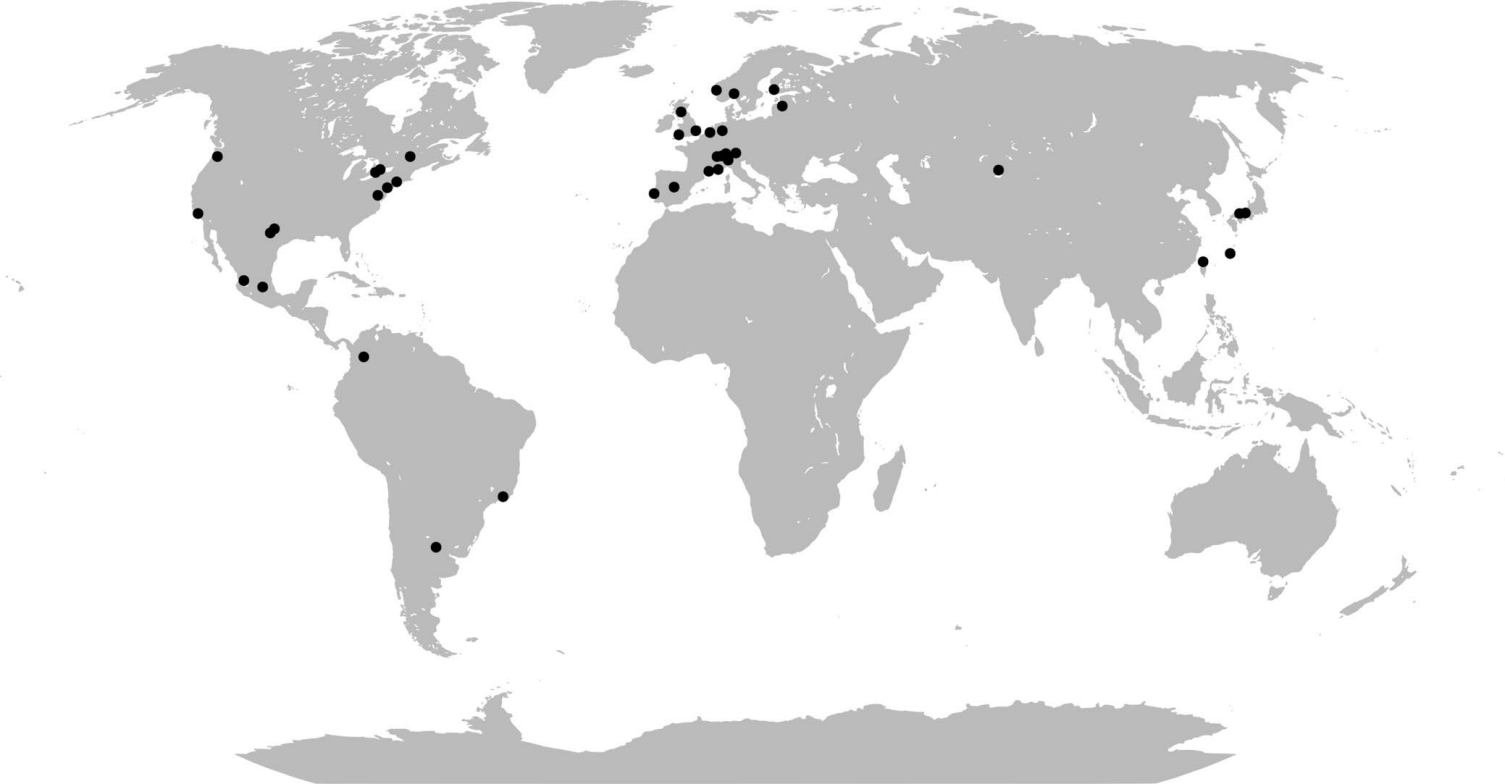
World map showing the locations of the 40 cities analyzed by our study. *Map source* Wikipedia.

We employ a comprehensive spatial analysis that integrates detailed geographical data on bike station locations with demographic data and population metrics across 40 global cities (see Fig. 1), representing a diverse range of geographical, economic, and cultural contexts. This approach does not only assess the physical coverage and distribution of bike stations, but also evaluates their accessibility in relation to the broader urban transit systems, providing a holistic view of their integration within each city’s unique urban fabric.

Moreover, our methodology enhances the understanding of how bike-sharing systems can be improved to serve larger urban populations effectively, focusing on accessibility and efficiency. By combining advanced spatial analytical techniques with extensive urban data, our research provides nuanced insights into the factors that contribute to the success and challenges of bike-sharing systems, proposing actionable strategies for urban planners and policymakers to enhance sustainable urban mobility. This study’s findings are poised to inform the development of more inclusive and effective bike-sharing systems that are better integrated with public transport networks, thus supporting the overarching goals of sustainable urban development.

## Methods

**Fig. 2:**
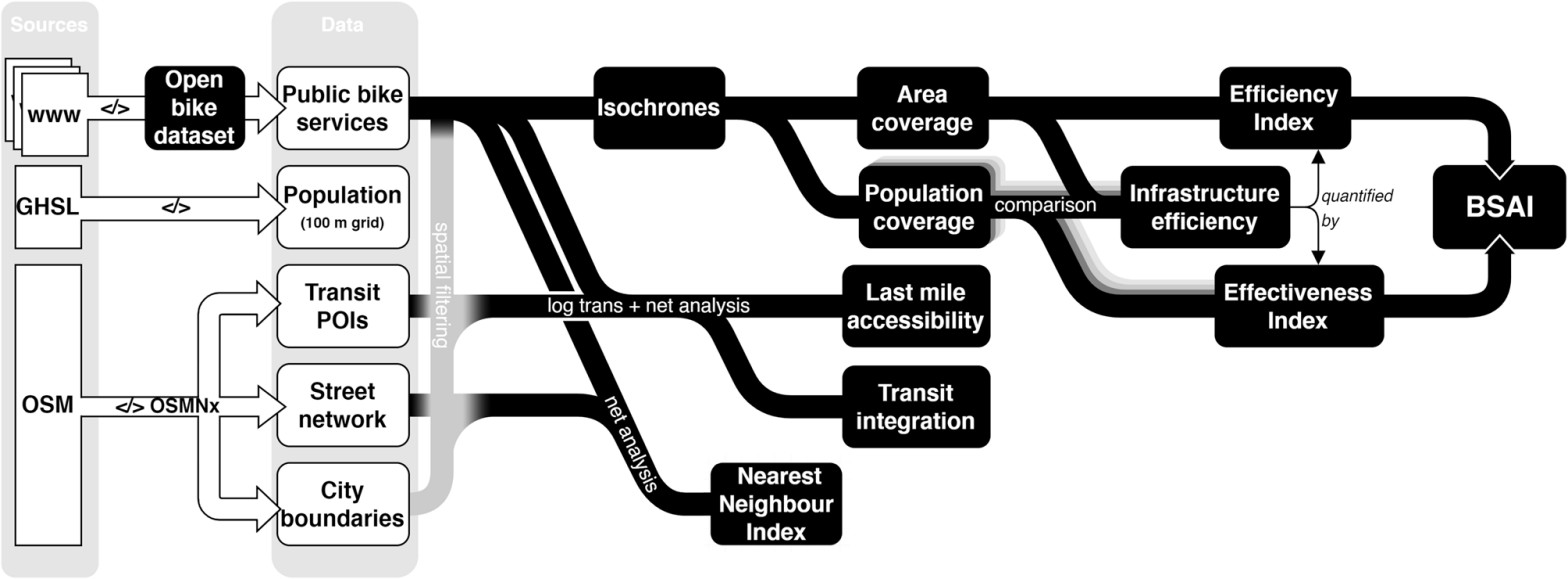
Workflow diagram illustrating the comprehensive methodology used to analyze bike-sharing stations in forty cities.

This section outlines the methodology employed to conduct a comprehensive analysis of bike-share stations across forty cities. Figure 2 illustrates the detailed workflow designed for this analysis. We discuss key components such as data sources, data acquisition processes, evaluation metrics, methods, scoring functions, and the scale of analysis. Over the years, various evaluation metrics have been proposed in the literature on bike-share analysis^38–40^. Our approach tailors these metrics to effectively assess and compare the bike-share networks, ensuring relevance and accuracy in our evaluation.

### Bike station data acquisition

To gather the data on bike share station locations, we relied on both city government websites and the Python library pybikes. Cities were primarily chosen based on the availability of open data. The primary source of information was the open data portals of various city governments, which provided detailed and accurate information about station locations. For some cities where data was not easily accessible through official channels, we used pybikes, which provided a standardized method of obtaining the data directly from the bike-sharing systems. This approach ensured that data from multiple sources could be combined consistently. To maintain consistency, all data was downloaded and updated until November 2023.

### Spatial distribution analysis of bike networks using the nearest neighbor index

In evaluating the spatial organization of bike sharing infrastructure within various cities, the Nearest Neighbor Index (NNI) can serve as a pivotal metric. This index quantifies the degree of clustering or dispersion among bike stations, offering insights into the distribution efficiency and accessibility of the network^41,42^.

The NNI is derived by comparing the *observed* mean distance to the nearest neighbor with the *expected* mean distance in a hypothetical random distribution. The formula for NNI is expressed as follows:

1 $$\begin{aligned} NNI = \frac{D_{obs}}{D_{exp}} \end{aligned}$$

Herein,

- $D_{obs}$, the observed average distance to the nearest neighbor, is calculated as
  2 $$\begin{aligned} D_{obs} = \frac{1}{n} \sum _{i=1}^{n} d_{i, min} \end{aligned}$$
  where $d_{i, min}$ is the distance from the *i*-th station to its closest neighbor;
- $D_{exp}$, the expected mean distance for a completely random distribution, is given by
  3 $$\begin{aligned} D_{exp} = \frac{1}{2\sqrt{\rho }}, \end{aligned}$$
  where
  4 $$\begin{aligned} \rho = \frac{n}{A} \end{aligned}$$
  represents the density of the points with *n* being the total number of stations and *A* being the area encompassed by the city boundary containing these stations.

The index yields a dimensionless value that categorizes the distribution of bike stations:

- a value < **1** indicates a clustered distribution, where stations are more closely spaced than expected by chance—such a clustering often reflects strategic placements within densely populated or high-demand urban areas;
- a value > **1** suggests a dispersed distribution, where stations are spread further apart than in a random layout, potentially indicating an expansion strategy to cover larger, less dense areas;
- a value $\approx$ **1** implies a random spatial distribution in the placement of bike stations.

Understanding the spatial distribution of bike-sharing stations through the NNI provides crucial insights into how these infrastructures integrate into the urban fabric. It helps identifying areas of potential over-saturation or under-service, which can guide strategic decisions regarding future expansions or adjustments in station placements.

### Population data acquisition

Population data is obtained from the Global Human Settlement Layer (GHSL). This data repository aims to provide global, uniform, validated, and semantic information, analytics, and knowledge about the human occupation on the entire planet at a fine resolution^43^. Particularly, the population data is extracted from the GHS-POP spatial raster dataset. This layer provides the estimation of the number of people living in each 100$\times$100 m cell in 5-year intervals from 1975 to 2030. The estimation uses ancillary data to improve the source raw global census data harmonized for the Gridded Population of the World (GPWv4.11)^44^. Particularly, for this research we used the base version with the coordinate system World Mollweide (ESRI: 54009) at 100 m resolution for the year 2023^45^. The 2023 release improves over previous versions by using independent data to correct estimates, harmonize coastlines, and double-check for unpopulated units. As a result, this dataset provides a homogeneous and accurate fine-grained description of residential population.

### Analyzing bike station accessibility and integration within urban transit systems

Access to key amenities such as public transportation, healthcare, education, and recreational facilities is essential for ensuring the livability, sustainability, and inclusivity of urban environments^46,47^. When addressing the critical concern of enhancing urban mobility, focusing in particular on the efficient integration of bike-sharing systems with established public transit networks, our methodology was underpinned by a multifaceted analytical approach. The core objective was to understand the extent to which bike-sharing systems complement existing public transit services and bridge the last-mile gap^48^, which is a pivotal element in urban planning known for its potential to significantly augment the utility and reach of public transit systems.

**Data acquisition and preprocessing** We utilized OpenStreetMap (OSM) to extract geographical information on public transit stops in 40 diverse cities. This data was accessed and handled using the osmnx Python library^49^, which facilitates the retrieval of detailed network data and geographical features from OSM. For each city under consideration, we delineated the urban boundary to filter the locations of interest—ensuring that our analysis was confined to the metropolitan area.

**Spatial analysis** Acknowledging the intricacies of spatial data, a pivotal aspect of our methodology revolved around the adoption of an appropriate Coordinate Reference System (CRS). To navigate the potential pitfalls associated with geographical distortions in distance calculations, we employed the Universal Transverse Mercator (UTM) projection system. A dynamic selection algorithm was implemented to assign the most fitting UTM zone based on the geographical centroid of each city. This strategic CRS alignment was instrumental in facilitating precise distance measurements, thereby enhancing the reliability of our spatial analysis.

**Median distance estimation** Central to our analysis was the determination of the median distance of bike-sharing stations to the nearest public transit stops within each city. This metric served as a quantitative gauge of the bike-sharing system’s integration with the public transit network, providing insights into the system’s efficacy in mitigating the last-mile problem. Through spatial joins and meticulous distance calculations within the harmonized CRS framework, we extracted these median distances, paving the way for a nuanced understanding of spatial accessibility across the selected cities.

**Distribution of distances** To gain deeper insights into the spatial relationship between bike stations and transit stops, we analyzed the distribution of distances in each city. Specifically, we analyzed the variability and density of these distances, identifying patterns and deviations that signify both optimal placements and areas necessitating strategic enhancements.

**Proximity to transit stops** Addressing the last-mile challenge necessitates ensuring that a substantial fraction of bike stations are within a convenient walking distance from transit stops. Defining a “convenient” distance as a 5-min walk^50^, assuming an average human walking speed, we determined the share of bike stations meeting this criterion. This analysis identified cities, where bike-sharing systems effectively complement public transit, facilitating easy access and encouraging multi-modal transportation.

Through this comprehensive approach, we aimed to elucidate the contributions of bike-sharing systems to urban transportation ecosystems. The findings from this analysis are intended to inform urban planners and policymakers, providing a data-driven foundation for strategic decisions aimed at enhancing urban mobility, accessibility, and sustainability.

### Data fusion, bike data, population data, and isochrones

**Fig. 3:**
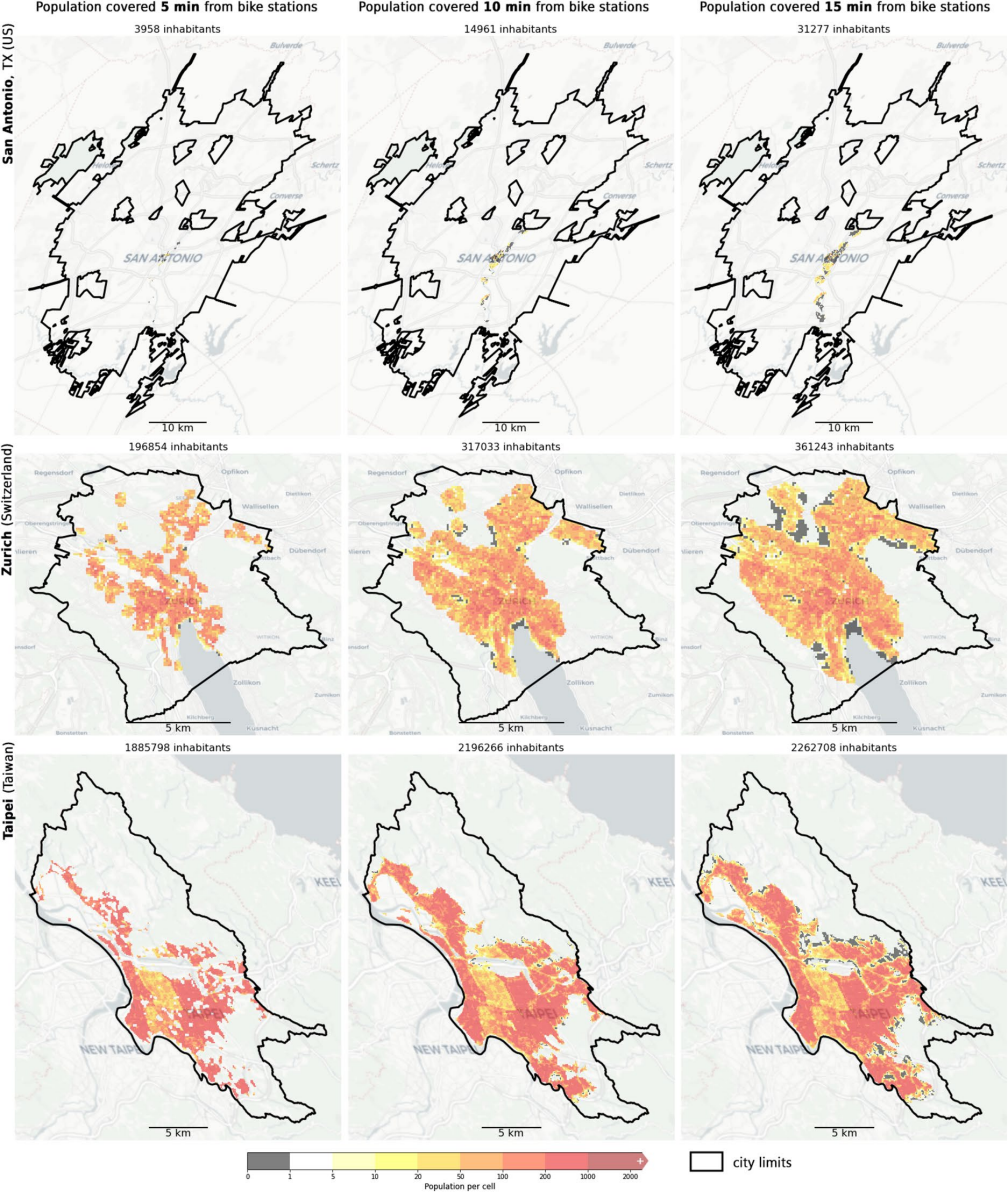
GHSL population raster cropped showing the coverage by the bike stations network for the 3 considered isochrone values (5, 10, and 15 min, respectively left, center, and right column) illustrated for 3 cities of the data sample. From top to bottom: San Antonio, Texas (US), Zurich (Switzerland), and Taipei (Taiwan). On top of each subpanel, the total population served by that isochrone is highlighted. The solid black line indicates the city limits as defined in OSM. Map sources: OSM^51^, CARTO.

We processed the GHSL world tiles for each analyzed city and cropped each raster based on the city limits as provided by Open Street Maps (OSM)^51^, using osmnx. While this approach may entail some limitations, as considering the official extent of cities does not necessarily accurately reflect the actual urban sprawl and its dynamics, it is a standardized procedure. Additionally, we masked out raster cells overlapping water areas, using OSM data tagged as natural=coastline. A comprehensive overview of this data is provided in Table S1in the Supplementary Materials. We computed the accessibility for each bike station using osmnx based on walking distances of 5, 10, and 15 min in the pedestrian network (network_type = “walk”)^49^, considering an average walking speed of 4.5 km/h, as discussed in previous studies^52,53^. Subsequently, we performed a Boolean union for all the resulting polygons corresponding to the same isochrone, in order to obtain full city coverage for 5, 10, and 15 min. These isochrone polygons are finally used to intersect with the raster and filter the overlapping cells. By summing up the values of the identified cells in the raster, the population effectively served by the bike station within the defined walking time is obtained, as shown in Fig. 3.

### Bike-share service accessibility index (BSAI)

We propose the Bike-Share Service Accessibility Index (BSAI), which is designed to offer a comprehensive evaluation of urban bike-share infrastructure, focusing not only on the extent and coverage but also on the effectiveness and efficiency of service delivery. The BSAI integrates multiple dimensions of bike-share infrastructure utility into a composite metric, enabling city planners and policymakers to gauge the performance of their bike networks more holistically.

*Methodology and rationale* The BSAI is calculated through a two-part process that assesses both the effectiveness and efficiency of bike-share infrastructure within urban environments. The effectiveness index (EI) measures the degree to which a bike-share infrastructure meets the coverage needs of the urban population and area. Meanwhile, the efficiency index (EFI) evaluates how well additional infrastructure investments translate into increased coverage. The BSAI is formulated as follows:

5 $$\begin{aligned} BSAI = \frac{EI + EFI}{2} \end{aligned}$$

Where:

- $EI = \frac{Served\_Pop\_Over\_Total + Served\_Area\_Over\_Total}{2}$ for the 15-min isochrone, representing the average of the population and area coverage ratios.
- $EFI = \frac{(Coverage\_15min - Coverage\_5min)}{2}$, where $Coverage\_15min$ and $Coverage\_5min$ represent the average of population and area coverage ratios at 15-min and 5-min isochrones respectively. This captures the change in coverage as the accessible range expands.

The inclusion of both population and area coverage in the EI allows for a comprehensive evaluation of bike-sharing systems across diverse urban contexts. While population coverage indicates service to residential areas, area coverage accounts for accessibility to key urban destinations that may have low residential density but high mobility demands, such as business districts, educational institutions, or recreational areas. This dual approach ensures that the index remains relevant for cities with varying urban forms and population distributions.

The formulation of BSAI captures both, the current state of infrastructure effectiveness and the incremental benefits of infrastructural enhancements. By employing a balanced approach (with equal weights for EI and EFI), the BSAI provides a unified metric that respects both the breadth of service provision and the strategic value of infrastructure expansion.

The BSAI is more than a mere evaluative tool; it can potentially be used for strategic urban planning and policy-making. A higher BSAI indicates not only extensive and effective bike-share infrastructure, but also efficient use of resources to expand service coverage. Conversely, a lower BSAI points to potential areas for targeted improvement. Specifically, the BSAI can inform:

- *Targeted investment for expansion* Areas with high EFI but low ES are prime candidates for infrastructural investment. These areas show potential for significant impact on coverage with additional resources, stressing the importance of strategic expansion to underserved regions.
- *Infrastructure upgrades* Conversely, low-performing areas (both in EI and EFI) highlight regions where both the quality and quantity of bike-share infrastructure may be lacking. Prioritizing these areas for upgrades can enhance overall network performance and user satisfaction.

By linking the BSAI to tangible planning outcomes, this metric becomes a pivotal tool in the development of more accessible and sustainable urban environments. It enables decision-makers to allocate resources more effectively, ensuring that bike-share infrastructure development aligns with broader goals of urban mobility, environmental sustainability, and public health.

## Results

### Interpretation of nearest neighbor index results

**Fig. 4:**
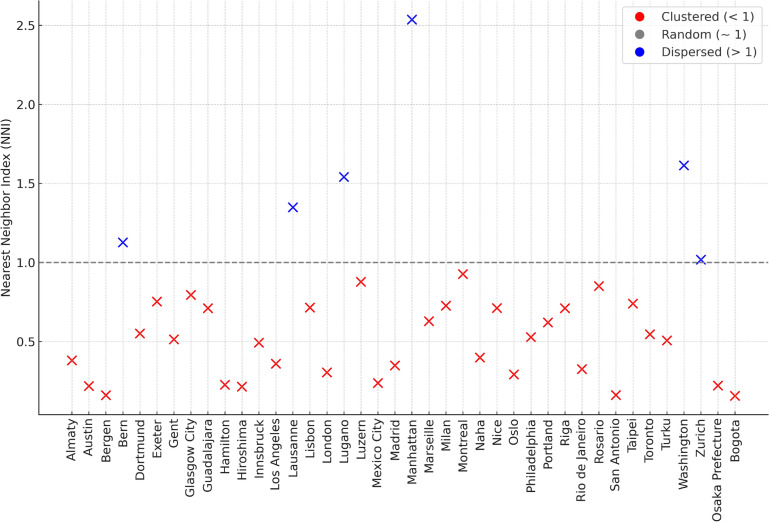
Nearest Neighbor Index for bike-sharing stations across selected global cities. This figure illustrates the spatial distribution patterns of bike-sharing stations, categorized by their degree of clustering.

The analysis of the Nearest Neighbor Index (NNI) values across cities reveals significant insights into the spatial distribution patterns of bike-sharing stations, which are indicative of differing urban planning strategies and geographic characteristics. Figure 4 displays these NNI values, categorizing the cities into three distinct groups based on their spatial distribution patterns: clustered, random, and dispersed.

Cities like Austin (0.219), Bergen (0.161), Almaty (0.381), and Los Angeles (0.36) exhibit a clustered distribution of bike-sharing stations. This suggests that these cities have strategically placed their stations in densely populated or high-demand urban areas. Such a strategy is often employed to maximize usage and ensure that a large number of potential users have easy access to the service. On the other hand, cities like Bern (1.127), Lausanne (1.349), Lugano (1.541), Manhattan (2.536), and Washington (1.614) show a dispersed distribution. This indicates an expansion strategy where stations are spread further apart to cover larger, less dense areas. This strategy could be employed to ensure wider coverage and accessibility across the city, including peripheral and less dense areas. Cities like Zurich (1.018) exhibit a random spatial pattern, suggesting no significant deviations from a random distribution in the placement of bike stations. This could indicate a more organic growth of the bike-sharing system, responding to a variety of factors such as user demand, availability of suitable locations, and city planning policies.

These findings provide valuable insights into the spatial strategies employed by different cities in the placement of bikesharing stations. They highlight the balance that needs to be struck between ensuring accessibility for a large number of users and providing wide coverage across the city.

### Integration of bike-sharing and public transit: a comparative analysis

**Fig. 5:**
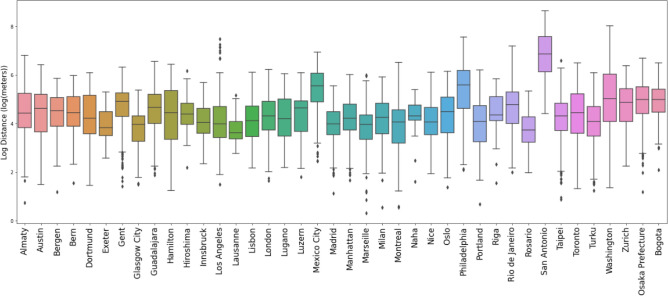
Analysis of urban bike station accessibility relative to public transit stops across various cities. The figure displays the log-transformed distances from the nearest public transit stop to bike stations across multiple cities, indicating the general proximity of bike-sharing infrastructure to essential public transit services.

The log-transformed box plot, as presented in Fig. 5, illustrates the distribution of distances between bike stations and public transit stops across cities on a logarithmic scale. This transformation mitigates the impact of extreme outliers, allowing for a more nuanced comparison of the distance metrics within and across the urban landscapes. Cities exhibiting lower median log-distances, such as Lausanne and Bern, reflect a dense and well-integrated network, where bike-sharing systems effectively complement public transit, offer robust last-mile solutions. Conversely, cities like San Antonio, which show higher median log-distances and wider interquartile ranges (IQR), may indicate a relative disconnect between bike station placements and transit stop accessibility. The log-scale underscores the variation in integration levels and provides a refined perspective on the urban mobility framework. It facilitates a comparative analysis that is less skewed by distance outliers, thereby supporting a better evaluation of the spatial dynamics at play.

The findings underscore the importance of strategic planning and investment in bike-sharing systems as part of a comprehensive public transport strategy. High accessibility to bike stations from transit stops not only addresses the last-mile challenge, but also fosters a sustainable transportation network that can reduce traffic congestion, decrease carbon emissions, and promote healthier lifestyles. For cities seeking to optimize their bike-sharing programs, these results highlight the necessity for data-driven and data-informed approaches in urban planning. By understanding the current landscape through spatial analysis, city planners can identify under-serviced areas, prioritize station placements, and ultimately cultivate more resilient and connected urban environments. In summary, these results suggest that the success of bike-sharing systems is not merely about the quantity of infrastructure but its thoughtful integration into the urban fabric.

**Fig. 6:**
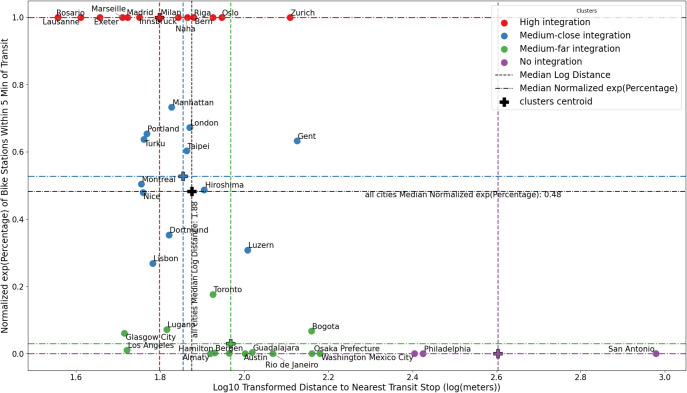
Relationship between bike-sharing station and existing public transit stops, based on the distance (*x*-axis) and percentage of bike stations within a 5 min walk from transit stations (*y*-axis). Four distinct clusters using KMeans are shown in different colors, together with the global (shown in black) and clusters’ centroids based on median values (shown in color).

New public bike rental systems can be considered new stand-alone micro-mobility solutions in the spirit of Mobility-as-a-Service (MaaS) or extensions of public transit networks. We can focus on how both variables are related (see Fig. 6), i.e. the distance to the closest transit station from a bike station on the one hand and the percentage of bike stations within 5 minute’s walk from a transit station on the other hand. This allows us to understand better how they are interrelated. Therefore, we used KMeans clustering and the Elbow and Silhouette methods to classify bike-sharing systems based on this relationship.

As a result, we can identify four main groups regarding their relation with existing mass transit services:

- *High integration* This is characterized by low distance values and a very high proportion of bike stations within 5 min of public transit stops. Bike service and public transit are connected and tend to cover approximately the same areas. As a result, they can operate as alternative systems, offering more options in case of congestion or disruption of service. This can be caused by an on-purpose design of the bike station networks to match public transit, or result from an already very good and dense coverage of public transit.
- *Medium-close integration* This is characterized by low to medium distance values with a high proportion of bike stations within 5 min of public transit stops. Public bike stations overlap to a large degree with the existing public transit services, and in some cases expand and complement their coverage. Hence, they could serve last-mile needs. A few bike stations are connected to existing public transit infrastructure and act as feeders for the rest of the bike stations that may be covering under-served areas of the city by mass transit.
- *Medium-far integration* This is characterized by medium to low distance values with a lower number of bike stations within 5 min of transit stops. There is a larger number of bike stations not coinciding with existing transit stops.
- *No integration* This is characterized by high distance values and a low proportion of transit stations near bike stations. Bike-share infrastructure and transit have a low level of interconnection. It can be caused either by low development of public transit in a city or by a localized or independent development of the bike-share infrastructure. It may also potentially the case in development phase of a bike service system.

Most of the European and Asian cities are located in the *high* and *medium-close* integration cluster. American cities tend to be more prevalent in the *medium-far* and *no integration* cluster. Even though the reason for this clustering may be associated with the currently prevalent types of urban planning and urban mobility patterns in each region, there are also differences between nearby cities. The urban conditions of each location and the design of its public transit determine the effectiveness and impact of similar bike-sharing systems. Therefore, a one-fits-all approach would not have the same performance in different cities.

From a qualitative point of view, this analysis allows us to put together the very different conditions of cities and their bike-sharing systems. Despite their differences, Mexico City and Philadelphia have common resulting features, which may help to design and test new policies. Conversely, the analyzed Swiss (i.e., Lausanne, Lugano, Luzern and Zurich) and Japanese (i.e., Hiroshima, Naha, and Osaka) cities, despite the common urban, cultural and policy characteristics, show very different interactions.

### Evaluating the efficiency of urban bike-share infrastructures

Here, we present two key aspects of public bike-share infrastructure across various cities: the average population coverage ratio and the average area coverage ratio.

**Fig. 7:**
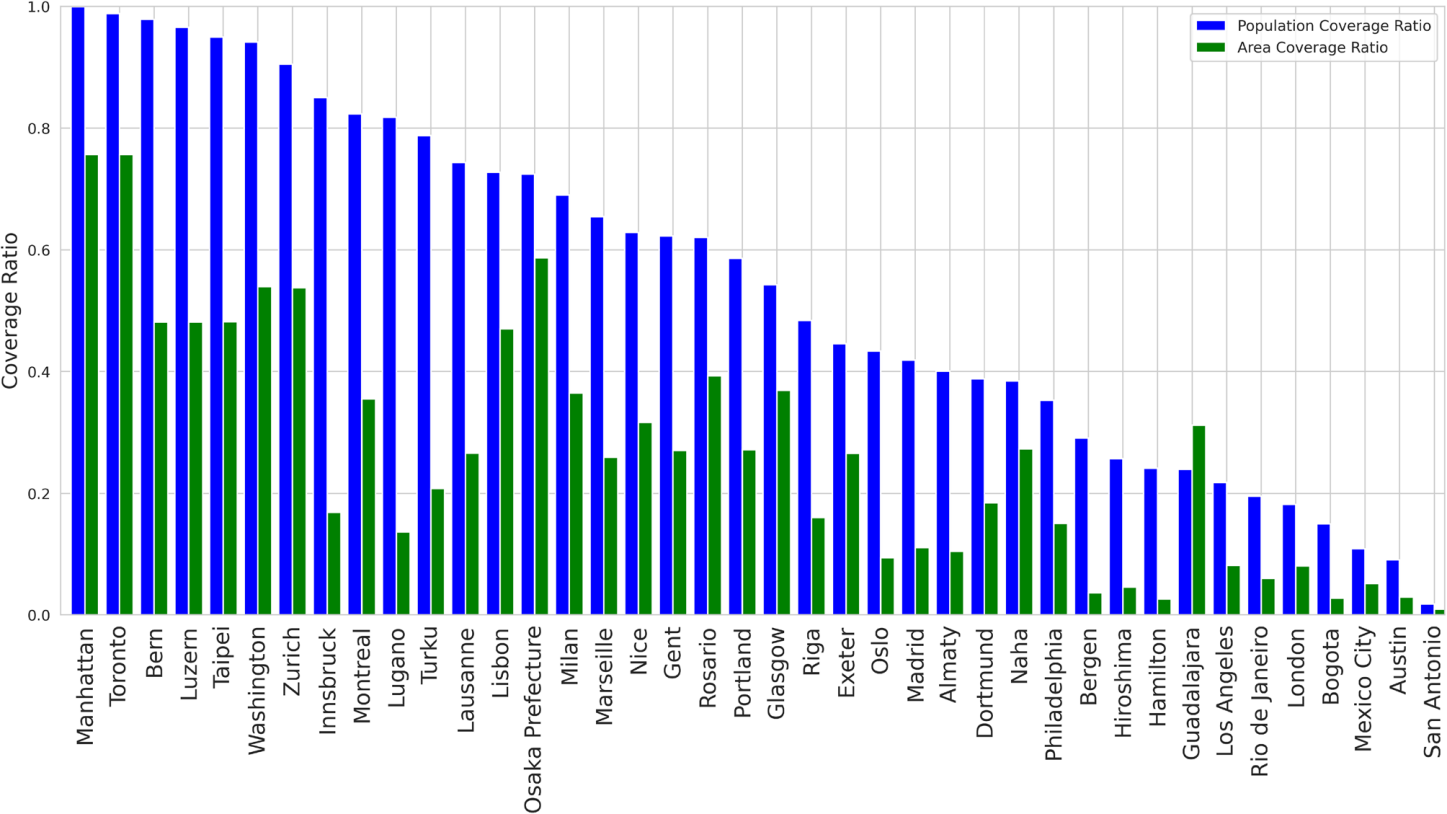
Analysis of bike sharing infrastructure coverage at a 15-min isochrone interval. The graph shows the average population coverage ratio and average area coverage ratio by city.

In our analysis of public bike-share infrastructure efficiency, we refined our analysis to concentrate on a standardized 15-min isochrone interval across 40 cities. This specific isochrone interval was chosen to represent a reasonable travel time for users, which can be a critical factor in the practical use of bike-sharing systems. In Fig. 7, we present a comparative analysis where we measure the extent of the population and urban areas within a 15-min radius of public bike stations. Figure 7 delineates cities according to their Population Coverage Ratio, where Manhattan stands out with the highest ratio, suggesting an exceptional integration of bike stations within dense population centers. Following closely are Toronto and Bern, both showcasing efficient designs of their bike networks in terms of accessibility. On the other hand, the Area Coverage Ratio highlights the physical spread of the bike networks across urban spaces. Manhattan and Toronto once again rank highly, indicating not only a focus on dense urban centers, but also a wider urban area reach, providing a comprehensive coverage that extends beyond the central city areas. This analysis allows us to identify which cities have optimized their bike-share infrastructure to serve both, a broad area and a significant proportion of their inhabitants effectively. Such insights are vital for urban planners and policymakers as they seek to enhance the usability and appeal of bike-sharing systems, ensuring they are a practical and integral part of urban mobility solutions.

**Fig. 8:**
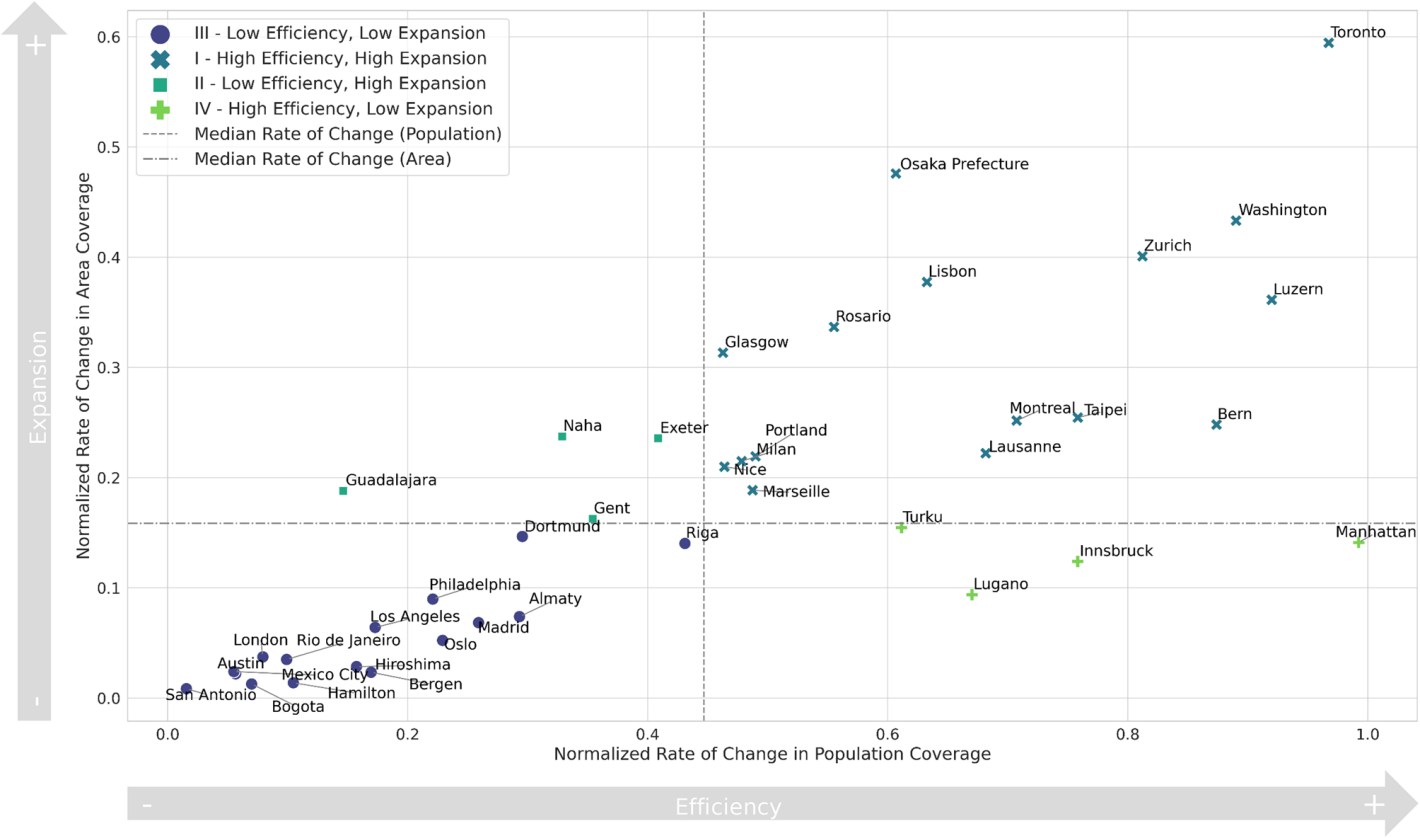
Rate of change in the 40 analyzed cities, considering the increase in the population served (x-axis) and covered area expansion (y-axis). As a result, four quadrants are identified, based on the median values of the change rates.

Next, we assessed the expansion of bike-share infrastructure in forty cities, focusing on the incremental changes in population and area coverage, as measured by isochrone intervals of 5, 10, and 15 min (Fig. 8). Understanding the incremental growth of bike-share infrastructure’s reach provides insights into each city’s approach to enhancing urban mobility. We initiated our analysis by identifying the initial coverage levels within a 5-min radius of bike stations, which were set as the baseline for subsequent evaluations. Each city’s potential for increasing its bike station coverage was quantified by calculating the maximum possible increase in population and area coverage, taking into account the under-served populations and areas within the 15-min isochrone.

The core of our method involved calculating the normalized rate of change between the 5-min and 15-min isochrones for both population and area coverage. This rate of change was defined as the ratio of the increase in coverage to the maximum possible increase, providing a standardized measure of expansion efficiency across the sampled cities. These calculations allowed us to observe not just the existence of infrastructure, but also to assess how effectively it is used to increase serviceable areas, thereby enhancing urban mobility.

Median values of these normalized rates of change were used to establish suitable thresholds to classify cities into four quadrants, reflecting distinct strategic approaches to infrastructure expansion. To establish thresholds for our classification, we employed median values due to their robustness against outliers and their capacity to provide a balanced split in a diverse dataset. The decision to segment cities into four distinct groups stems from our aim to offer a nuanced yet interpretable classification that effectively addresses both the extent of area coverage and the population served by bike-share systems. This method allows for a multi-dimensional analysis, which is crucial for evaluating the performance of bike-share systems across varied urban environments.

The four quadrants are defined as follows:

- *High efficiency, high expansion* Cities that showed significant improvements in both population and area coverage.
- *Low efficiency, high expansion* Cities that expanded their geographical coverage significantly without a corresponding increase in population coverage efficiency.
- *Low efficiency, low expansion* Cities that achieved a limited growth in both dimensions, indicating either a possible saturation or some strategic challenges.
- *High efficiency, low expansion* Dense urban centers, where significant population coverage was reached within a short range, but with little additional geographical expansion.

Our findings are shown in Fig. 8 and present a nuanced picture of the development of urban bike-sharing systems. The main sequence associates the increase of spatial coverage and a larger population served by bike-sharing services with performing over and below the median as shown by quadrants I and III respectively. While cities in quadrant III show a stronger association between geographical coverage expansion and increase of population served, this becomes less apparent for the other cases. Particularly interesting are the cities situated in quadrant II that show a very efficient expansion in the population served with a smaller coverage expansion. That can be due to a number of factors, such as high population density, geographical, physical, and topographic constraints that limit the spatial expansion of the coverage, and the strategic placement of stations in highly populated areas. Two noteworthy outliers that illustrate these cases are Lugano and Manhattan. Lugano shows a complicated topography that conditions the expansion of the built-up area and bike-sharing stations, while simultaneously it is densely populated. Similarly, Manhattan presents a very high density of population with a coverage of bike-sharing stations that quickly fills the whole available space of the island. Differently, quadrant IV shows exactly the opposite trend: it represents bike stations that, despite geographical expansion, do not manage to serve the population efficiently. This may be caused by urban sprawl and overall low population density or by choosing bike station locations in zones that are not densely populated. Another factor that may affect the expansion of the geographic coverage is the closeness or concentration of stations. Some of the cities in quadrant III may exhibit a development of bike-sharing services that prioritize a redundant coverage, where stations overlap within 15 min walking distance, instead of prioritizing expansion schemes such as in the case of Quadrant I.

By focusing on relative changes and utilizing normalized rates of change, our analysis accounts for diminishing returns of service expansion in areas, where the bike-share infrastructure is already good. Thus, our findings shed light on how cities are developing their bike networks not just in size but also in scope and serviceability, reflecting a deeper commitment to the ideals of accessibility and sustainability increasingly championed by urban development policies.

### Analysis of urban bike-share infrastructure through BSAI

Following the exploration of population and area coverage ratios and the examination of relative expansion rates within urban bike-share infrastructures, we introduced the BSAI as a novel metric to further elucidate the performance of bike networks across 40 cities. Through the BSAI, we have sought not only to measure the physical reach of bike-sharing systems, but also to assess the strategic effectiveness of their distribution and the efficiency with which they extend their service to under-served areas. This dual focus provides a comprehensive picture of how well cities leverage their bike-share infrastructure to foster inclusive, accessible, and sustainable urban environments.

**Fig. 9:**
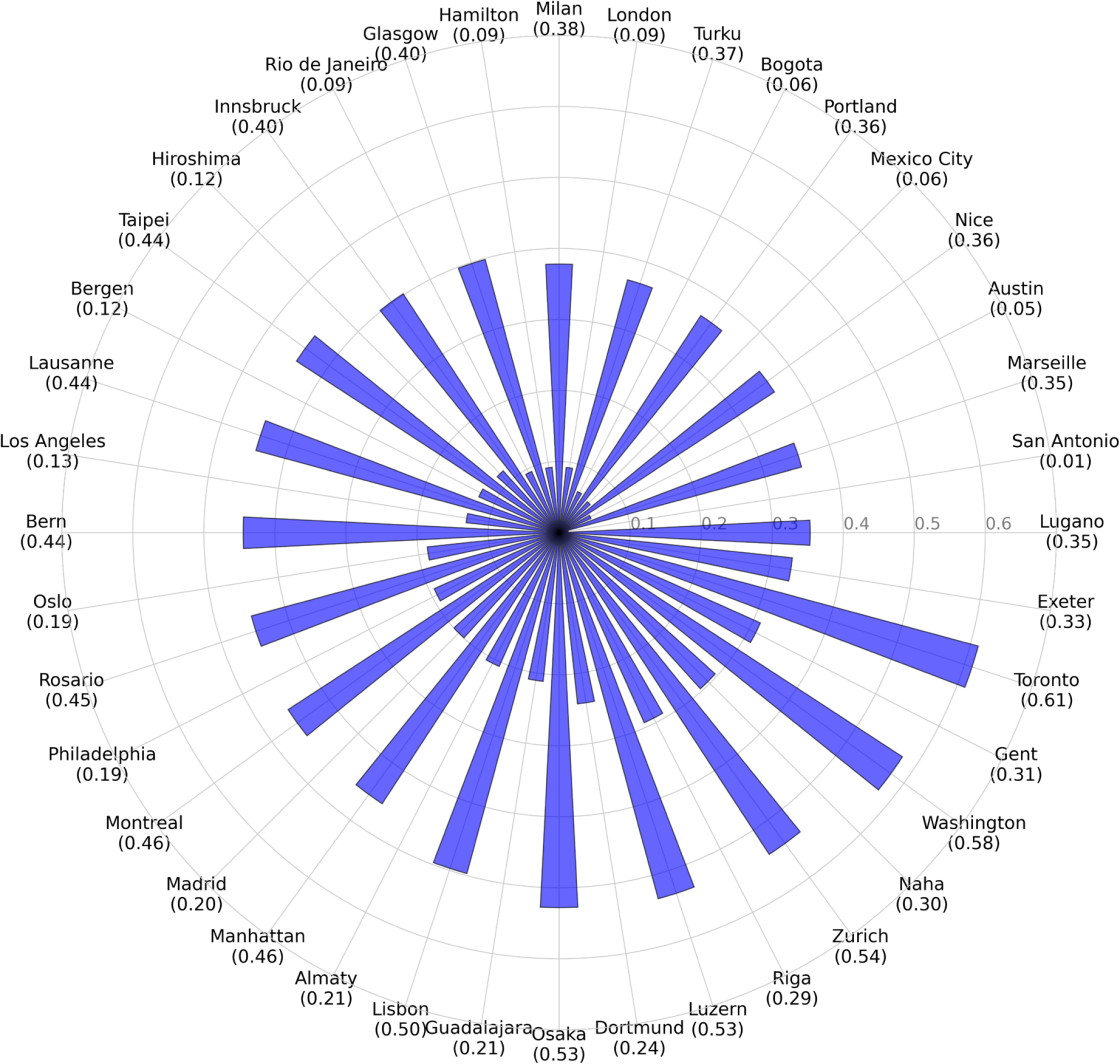
Bike-Share Service Accessibility Index (BSAI) across selected cities. The index provides a comparative overview of the effectiveness and efficiency of bike-share infrastructures, reflecting both, the current performance and the potential for future expansion.

The BSAI findings, illustrated in Fig. 9, give a differentiated picture of urban mobility and planning. Cities like Toronto, Washington, and Zurich stand out for their exemplary bike-share infrastructures, reflecting a concerted effort to integrate bike-sharing systems seamlessly into their urban mobility network. These cities have not only achieved extensive coverage, but also ensured that their infrastructure effectively meets the diverse mobility needs of their populations. Such achievements underscore the potential of bike-sharing systems to significantly contribute to the urban transportation ecosystem, when strategically aligned with broader mobility and sustainability goals.

In contrast, the lower BSAI observed in cities such as Austin and San Antonio reveal critical gaps in bike-share infrastructure provision and utilization. These gaps highlight opportunities for targeted interventions to enhance both the reach and impact of bike-sharing systems. By identifying specific areas, where bike-share infrastructure lags in effectiveness and efficiency, the BSAI directs attention to where strategic investments and planning initiatives can achieve substantial improvements in urban mobility.

## Discussion

### Implications of results

The creation of a comprehensive dataset of bike-sharing systems in different cities around the globe allowed us to compare them according to a set of criteria. Our results are valuable as they allow for a nuanced understanding of the development strategies and underlying criteria and policies guiding bike-sharing services. By analyzing bike-share infrastructure, our study provides a streamlined and clear comparison of bike-sharing services across cities. This approach offers valuable insights into the distinctive characteristics and varied implementation models that influence accessibility across different urban contexts.

The classifications shown in Fig. 8 highlight the various strategies that cities may implement. For instance, cities in quadrant III (low efficiency, low expansion) may be focusing on developing robust bike-sharing systems with overlapping stations within a 15-min walk radius, providing alternatives to existing public transit and additional mobility options for the served area. In contrast, cities in quadrant I (high efficiency, high expansion) demonstrate a balanced approach of expanding both the service area and population served, probably prioritizing expansive networks to enhance last-mile mobility from existing public transit services. The results depicted in Fig. 6 underscore the importance of considering bike-sharing infrastructures as an integral part of a city’s transportation system. This classification offers insights into how well these systems are integrated with public transit.

However, it is essential to recognize that the development of public transit systems varies significantly across the globe, which may also explain some observed differences. This is not solely attributable to the bike-sharing systems. Additionally, some urban structures may be more conducive to expansive schemes that efficiently increase the population served. By understanding these variations, urban planners and policymakers can better tailor bike-sharing strategies to fit the unique characteristics of each city, ultimately contributing to more effective and inclusive urban mobility solutions.

**Fig. 10:**
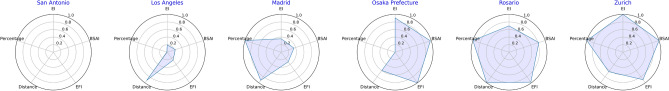
Analysis metrics (EI, BSAI, EFI, percentage bike sharing stations within 5 min of transit stops, and the average distance from bike sharing stations to nearest transit stop) of bike sharing services in selected cities, which feature distinctive characteristics. San Antonio is an example of a bike sharing services with limited scope. Los Angeles shows an early development that seems to prioritize connectivity to existing transit. Madrid features a high level of integration with public transit while Osaka feature an extensive bike sharing service. Rosario and Zurich feature simultaneously extensive and well integrated networks. For data of all the analyzed cities, see Figs. S2 and S3 in Supplementary materials.

Our analytical framework focuses on two main aspects: (i) a spatial dimension connected to population and geographic scope represented by the BSAI, EI and EFI metrics, and (ii) a transportation dimension based on how it related to existing transit services shown by the metrics of percentage stations within 5 min of transit and the distance to nearest transit stop. This framework allows us to compare different networks under uniform parameters. Hence, it is possible to identify distinctive stages, strategies and configuration of bike sharing services. In Fig. 10 we illustrate some very distinctive situations. For instance, San Antonio features a limited scope of its bike sharing service, while Los Angeles early development seems to have a focus on connectivity with existing transit. Madrid features a bike sharing network that has prioritized a strong integration with existing public transit, while Osaka has focused on network geographical expansion across the city. A few other cities such as Rosario or Zurich show a balanced network on both dimensions.

### Limitations and future work

While the selection of the 40 studied cities was diverse, there is still a lack of available data for African and Asian cities, probably due to limited bike-sharing infrastructures and/or lack of open data^54,55^. Our analysis focused on population density, but we acknowledge that other factors such as city shape and topology, land use, socioeconomic differences across neighborhoods, etc. may also play a crucial role for the expansion, development, and coverage of bike-sharing infrastructure. To ensure our analysis was consistent and comparable, we focused on using complete and uniformly available data for all 40 cities. This approach allowed us to apply a standardized methodology across diverse urban contexts. Future research could consider incorporating additional factors, when more comprehensive datasets become available, thereby further enhancing the understanding of bike-sharing network dynamics.

Factors such as the number of users, subscription costs, terms of use (including maximum trip durations), and the types of bikes available (e.g., electric, regular, cargo) are crucial for a comprehensive understanding, and are areas for future research. It’s important to note that our analysis focuses solely on station-based bike-sharing systems. We acknowledge that in some of the studied cities, dockless bike-sharing, e-scooters and free-floating bike services may coexist with the station-based systems. These dockless services could potentially influence the usage patterns and coverage of station-based systems. However, due to the lack of consistent and reliable data on dockless services across all 40 cities, and the often-changing nature of these services, they were not included in our analysis. Future research could explore the interplay between station-based and dockless micro-mobility services to provide a more comprehensive picture of urban mobility options. Incorporating spatio-temporal data, such as the number of bikes available at each station, station capacity, and origin-destination matrices, would provide an even more detailed picture of system usage. Unfortunately, such detailed data is currently not accessible for most cities included in our study.

The interpretation of our results should not be viewed as a definitive assessment of the quality of bike-sharing services in each city. Such an analysis would require additional data, including user satisfaction and the interplay within the general mobility patterns of each city. Despite these limitations, our analysis allows for meaningful comparisons of bike-sharing systems across diverse urban contexts, using common characteristics collected from all analyzed locations. By focusing on uniformly available data, we were able to conduct a robust and consistent analysis across a diverse set of cities, laying the groundwork for more detailed future studies that can build upon our findings as more data becomes available.

## Conclusion

This study provides a foundational analysis of bike-sharing systems across 40 global cities, offering insights into their spatial distribution, integration with public transit, and overall effectiveness. The introduction of the BSAI and the use of advanced spatial analysis techniques contribute to a deeper understanding of the role of urban bike-share infrastructures for sustainable mobility.

The key take-aways of this work are:

- *Spatial distribution patterns* Cities exhibit varied spatial distribution patterns of bike-sharing stations, reflecting different urban planning strategies and geographic characteristics.
- *Integration with public transit* The degree of integration between bike-sharing systems and public transit varies significantly across cities, impacting the overall efficiency and accessibility of urban mobility networks.
- *Effectiveness and efficiency* The BSAI provides a comprehensive metric for evaluating the performance of bike-sharing networks, highlighting areas for improvement and investment.

Future studies could build on these findings by incorporating more diverse data and exploring urban dynamics more broadly. Including underrepresented regions, integrating more detailed socio-economic and spatial data, and examining the impact of different bike types (e.g. eBike or not) as well as operational models will provide a more comprehensive understanding of urban bike-sharing systems.

## Supplementary Information

Supplementary Information.

## Data Availability

To ensure the transparency and reproducibility of our research, we will make all the data, shapefiles, and code publicly available after acceptance via an open-access GitHub repository. The working repository can be accessed using this link: https://github.com/sachit27/Bike_Accessibility.
