# Supplementary material for: Global comparison of urban bike-sharing accessibility across 40 cities
Source: Sci Rep. 2024 Sep 3;14:20493. doi: 10.1038/s41598-024-70706-x (PMC11379918; doi:10.1038/s41598-024-70706-x)
Supplement: Supplementary file 1 — Supplementary Information. [file 41598_2024_70706_MOESM1_ESM.pdf]

Supplementary Information

Global Comparison of Urban Bike-Sharing Accessibility Across 40 Cities

Authors and Affiliations: Sachit Mahajan[1,*], Javier Argota Sánchez-Vaquerizo[1]

[1] Computational Social Science, ETH Zurich, 8092 Zürich, Switzerland

[*] Corresponding Author: sachit.mahajan@gess.ethz.ch

SUPPLEMENTARY TABLE

Supplementary Table S1. Overview of bike-sharing systems in studied cities, including the number of bike stations and city population.

City	Number of Bike-Share Stations	Population
Almaty	176	2153172
Austin	79	1168917
Bergen	120	286137
Bern	192	123805
Bogota	296	9146543
Dortmund	96	608739
Exeter	16	122291
Gent	437	260277
Glasgow	107	613951
Guadalajara	227	1363704
Hamilton	144	560687
Hiroshima	139	1116082
Innsbruck	48	143502
Los Angeles	220	3919902
Lausanne	17	139685
Lisbon	152	571182
London	795	9357594
Lugano	38	65435
Luzern	85	84311
Mexico City	491	8014227
Madrid	235	3686229
Manhattan	643	1616537
Marseille	178	847075
Milan	318	1261295
Montreal	730	1755809
Naha, Okinawa	26	325995
Nice	136	305214
Osaka	426	2523228
Oslo	247	675555
Philadelphia	232	1580652
Portland	236	670103
Riga	42	616193
Rio de Janeiro	370	6877535
Rosario	86	997296
San Antonio	32	1752685
Taipei	1186	2382786
Toronto	688	796714
Turku	222	184718
Washington	374	717208
Zurich	155	399230

SUPPLEMENTARY FIGURES

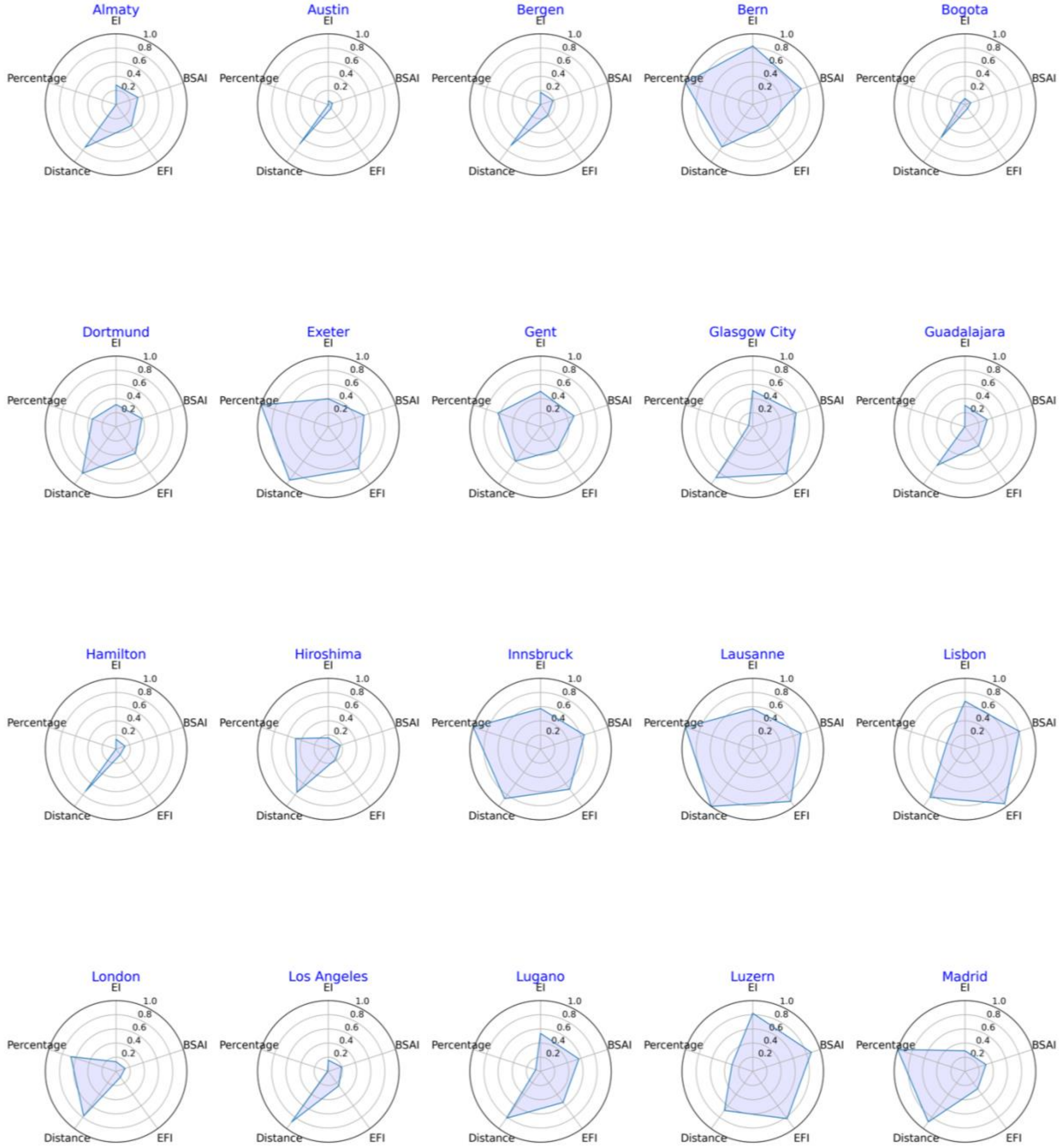

Supplementary Figure S2. Analysis metrics (EI, BSAI, EFI, percentage of bike-sharing stations within 5 minutes of transit stops, and the average distance from bike-sharing stations to nearest transit stop) of bike-sharing services for all cities (part 1).

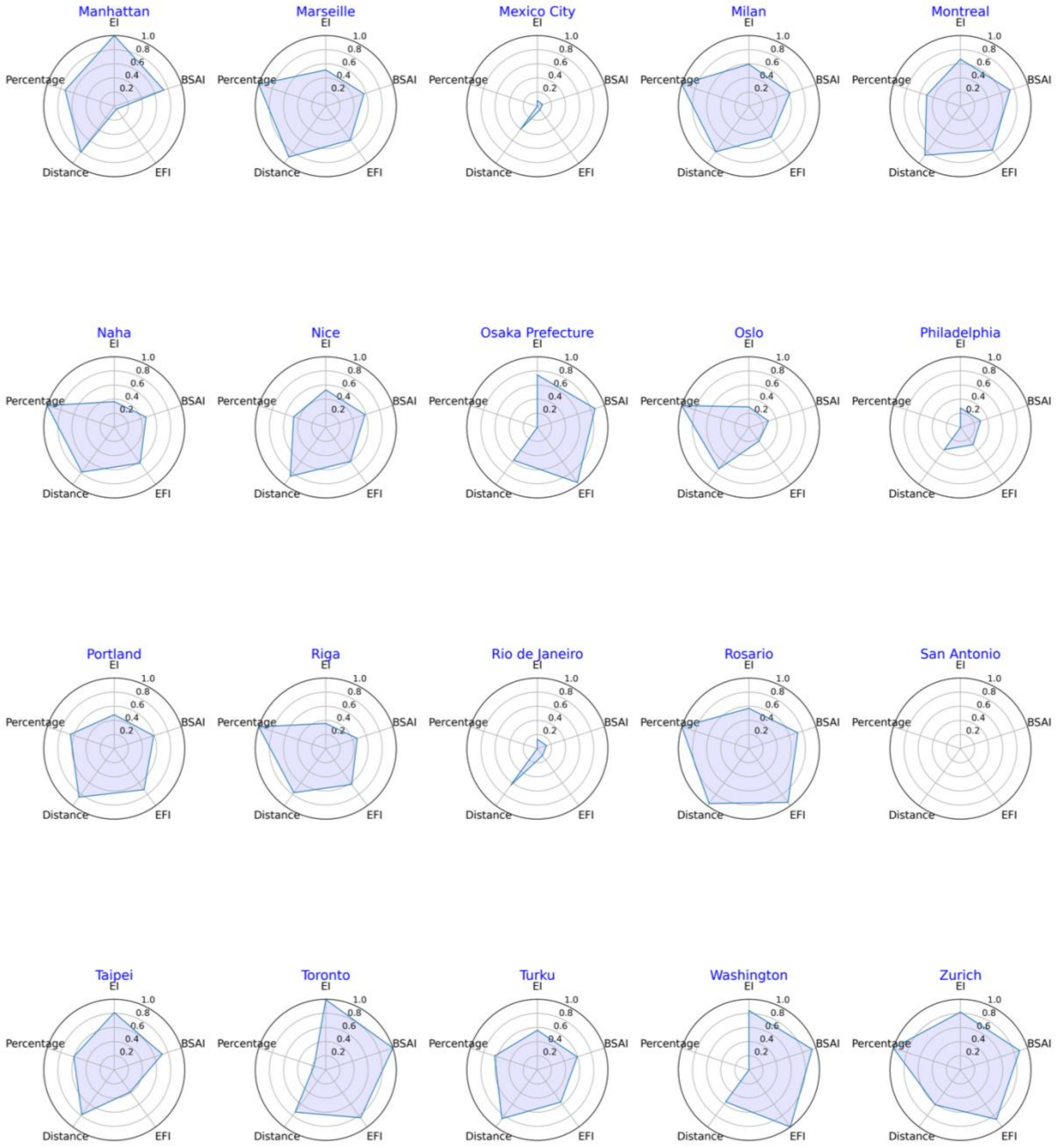

Supplementary Figure S3. Analysis metrics (EI, BSAI, EFI, percentage of bike-sharing stations within 5 minutes of transit stops, and the average distance from bike-sharing stations to nearest transit stop) of bike-sharing services for all cities (part 2).
